# Compositional Constraint Is the Key Force in Shaping Codon Usage Bias in Hemagglutinin Gene in H1N1 Subtype of Influenza *A* Virus

**DOI:** 10.1155/2014/349139

**Published:** 2014-07-17

**Authors:** Himangshu Deka, Supriyo Chakraborty

**Affiliations:** Department of Biotechnology, Assam University, Silchar, Assam 788011, India

## Abstract

It is vital to unravel the codon usage bias in order to gain insights into the evolutionary forces dictating the viral evolution process. Influenza *A* virus has attracted attention of many investigators over the years due to high mutation rate and being cross-specific shift operational in the viral genome. Several authors have reported that the codon usage bias is low in influenza *A* viruses, citing mutational pressure as the decisive force shaping up the codon usage in these viruses. In this study, complete coding sequences of hemagglutinin genes for H1N1 subtype of influenza *A* virus have been explored for the possible codon usage bias acting upon these genes. The results indicate overall low bias with peaking ENC values. The GC content is found to be substantially low as against AT content in the silent codon sites. Significant correlations were observed in between the compositional parameters versus AT_3_, implying the possible role of the latter in shaping codon usage profile in the viral hemagglutinin. The data showed conspicuously that the sequences were *A* redundant with most codons preferring nucleotide *A* over others in the third synonymous codon site. The results indicated the pivotal role of compositional pressure affecting codon usage in this virus.

## 1. Background

Influenza* A* virus (IAV), a member of* Orthomyxoviridae,* remains a serious health concern on a global basis with a number of epidemics since early 19th century till date. With several variants of varying pathogenic profile, IAV is causing significant mortality every year throughout the globe. In the year 2009, the world has seen its only second global pandemic, an H1N1 pandemic which was declared as phase 6 alert level by the World Health Organization (WHO). It was the first of its kind since 1968 when Hong Kong flu was declared a global pandemic by the WHO. Reports say that about 214 countries have been affected by the pandemic influenza H1N1 of 2009 taking 18,138 lives, as updated in May 2010 (http://www.who.int/csr/don/2010_06_04/en/index.html).

What makes influenza* A* such a deadly virus? Generally, upon exposure to a pathogen, the host develops specific immunity against it, thus, preventing the same pathogen infecting for a second time. The IAV escapes the specific immunity of the host by a process termed as antigenic drift. This is achieved by frequent mutation in the hemagglutinin (HA) and neuraminidase (NA) genes which encode the main antigenic determinant proteins in the virus, due to which immunogenically distinct strains develop which cause the seasonal outbreaks [1]. Another process, differently termed by different authors as cross-specific shift [2] or reassortment [3], is responsible for the frequent changes in the antigenic region of the virus, as happened in case of 2009 H1N1 pandemic. The viral HA or NA or other gene segments of different subtype of IAV are exchanged resulting into a novel subtype of IAV. These two genes, HA in particular, provide virulence to the virus making it as a potential drug target for the prevention of the spread of influenza infection [1].

The degeneracy of the genetic code has rendered the privilege of using more than one codon to code for the same amino acid. The phenomenon is called synonymous use of codons. The use of synonymous codons, however, is not uniform in different species ranging from prokaryotes to complex organisms as well as in viruses; certain synonymous codons are used preferentially. This tilted use of codons is termed as codon usage bias (CUB). With the rapidly growing stockpile of sequences in public databases after whole genome sequencing of large number of species, investigators have engaged in research in the context of codon usage bias in specific genes as well as whole genome of a vast range of organisms [4–7].

The preferential use of synonymous codons is governed by different evolutionary forces [8]. Over the years many authors have reported a number of measures to assess codon usage bias across genes and genomes. Among these measures, GC content, relative synonymous codon usages (RSCU), and effective number of codons (ENC), are some of the most widely used parameters for codon bias study. Much has been debated regarding the inclination towards the selection of optimal codons in genes; many advocated increased efficiency of translation process as the main reason behind selection of optimal codons [9]. However, the exact mechanisms behind synonymous codon variation are yet to be understood clearly.

Several workers have reported that the overall codon usage bias in RNA viruses is low, which is attributed to GC compositional properties and dinucleotide content in these viruses [5, 10–12]. Mutational bias has been projected as the main factor that drives the codon usage variation among the influenza* A* viruses which are phylogenetically conserved [10, 12, 13].

## 2. Materials and Methods

### 2.1. Datasets

In this study, a total of 32 complete coding sequences of the hemagglutinin (HA) gene of human-host derived influenza* A* virus subtype H1N1 reported from India were retrieved from NCBI (http://www.ncbi.nlm.nih.gov/). The serial numbers (SN), accession numbers, and other information are presented in supplementary Table 1 available online at http://dx.doi.org/10.1155/2014/349139.

### 2.2. Parameters for Codon Usage Bias Study

Relative synonymous codon usage (RSCU) [14] is one of the most widely used parameters for querying the pattern of synonymous codon usage across genes and genomes without confounding influence of the amino acid composition. To examine the synonymous codon usage in the genes, RSCU values were calculated. RSCU is defined as the ratio of the observed frequency to the expected frequency if all the synonymous codons for those amino acids are used equally. If the RSCU value of a codon is more than 1.0, it is said to have a positive codon usage bias, while a value of less than 1.0 means a negative codon usage bias. When the RSCU value is close to 1.0, it means that this codon is chosen randomly and equally with other synonymous codons.

The effective number of codons (ENC) estimates the enormity of codon usage bias in a gene [15]. ENC is estimated to quantify the synonymous codon usage across the target sequence which is calculated as given below:

(1) $$\begin{array}{c} \text{ENC}=2+\frac{9}{F_{2}}+\frac{1}{F_{3}}+\frac{5}{F_{4}}+\frac{3}{F_{6}}, \end{array}$$

where, *F*_*k*_  (*k* = 2,3, 4  or6) is the average of the *F*_*k*_ values for *k*-fold degenerate amino acids. The *F* value denotes the probability that two randomly chosen codons for an amino acid with two codons are identical. The values of ENC range from 20 (when only one codon is used per amino acid) to 61 (when all synonymous codons are equally used for each amino acid) [15–17]. The codon bias is considered low if the ENC value is greater than 40.

Nucleotide composition plays a crucial role in the codon usage pattern in the genes because most of the indices of codon usage bias are based on the base composition of the genes. GC_3_ is the frequency of the nucleotides G+C at the synonymous 3rd positions of the codons excluding the* Met*,* Trp*, and the termination codons. Similarly, GC_1s_ and GC_2s_ represent G+C frequency at 1st and 2nd codon positions. GC_3s_ is a good indicator of the extent of base composition bias.

Gene expressivity was measured by codon adaptation index (CAI) as given by Sharp and Li [14]. CAI has been used as a simple and effective parameter to measure the adaptiveness of synonymous codon usage bias of a gene towards the codon usage of highly expressed genes. CAI, with the boundary values 0-1, was originally proposed to provide a normalized estimate that can be used across genes and species. A value of 1 is assigned to the most frequent codons within a gene (CAI = 1) while the least frequent codons are assigned a CAI value of 0 [18, 19]. CAI is estimated as

(2) $$\begin{array}{c} \text{CAI}=\exp\frac{1}{L}\overset{L}{\underset{k=1}{\sum }}\ln w_{c(k)}, \end{array}$$

where *L* is the number of codons in the gene and *w*_*c*(*k*)_ is the *ω* value for the kth codon in the gene.

Frequency of optimal codon (Fop), originally proposed by Ikemura in the year 1981, is one of the first estimators used in the study of codon usage bias. As an index, Fop shows the optimization level of synonymous codon choice in each gene to translation process [8]. Fop is defined as the ratio of total number of optimal codons in a gene to the total number of synonymous as well as nonsynonymous codons in that gene.

The codon usage bias measures, namely, RSCU, ENC, GCs, Fop, and CAI for each coding sequence, were estimated in our study by using an in-house Perl program developed by SC.

## 3. Results and Discussion

### 3.1. Nucleotide Compositional Properties

The coding sequences were analyzed thoroughly for their nucleotide composition. Individual nucleotides as well as GC and AT content in three synonymous codon positions were estimated. The nucleotide composition in the analyzed genes is summarised in Table 1. The results reveal that the viral hemagglutinin is* A* redundant with overall* A* content of 35.3% with a range of 34.9% to 35.6% and standard deviation (SD) of 0.167. On the other hand, the *C* content in all the accessions is consistently low ranging from 18.2% to 18.8% with average and SD of 18.5 and 0.145, respectively.

The frequency of codons containing dinucleotide TpA is much higher in comparison to those containing dinucleotide CpG. Four codons, that is, CGA, CGC, CGG, and CGT, out of possible nine codons containing CpG, are absent in the analyzed gene; the frequencies of the remaining codons are also very low with the highest value of 9 for GCC. In contrast, most of the codons (5 out of 6) containing TpA showed higher frequency with the highest value of 17 for GTA and the lowest 6 for TTA. While three codons containing TpA are preferred, there are no preferential codons containing CpG.

**Figure 1:**
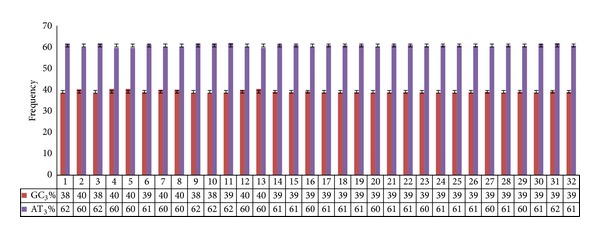
Comparison of AT and GC content at synonymous third codon positions in the genes under study. Clearly, AT_3_ is much higher than GC_3_ in all the accessions.

The overall GC content in the dataset was found to be much lower in comparison to overall AT content (40.7% and 59.3%, resp.). The suppression of GC content as compared to AT content is also evident from GC/AT content at the silent position. The overall GC_3_ was found to be low (39.0%) as against AT_3_ (60.7%) (Figure 1). To detect any possible relation of base composition at different synonymous codon positions, the estimated values of the four nucleotides *A*, *T*, *G*, and *C* and the AT and GC content were compared with the values of the nucleotides in third synonymous positions (i.e., *A*_3_, *T*_3_, *G*_3_, and *C*_3_). The results indicate a strongly significant and complicated correlation which is presented in Table 2. The correlation coefficients were highly significant in majority of the parameters taking both positive and negative values except a few showing insignificant correlation. Negative correlation was also observed between GC_1+2_ and GC_3_  (*r* = −0.478, *P* < 0.001). The correlation results indicate the possible role of mutational pressure acting on these genes. The base composition was most likely influenced by AT_3_ as revealed by the highly significant correlation coefficients.

Previous studies have revealed that the CpG underrepresentation is attributable to immunologic escape, in order to avoid host immune system using the unmethylated CpGs as a pathogen marker [20, 21]. CpG deficiency has also been reported in some other RNA viruses as well [10, 20, 22]. Thus, combating the host immune response may constitute a selection pressure in these viruses.

The general trend of the ENC values suggests the absence of strong codon bias in the hemagglutinin gene. The ENC values were consistently found in higher range with an average value of 58 ± 0.363. Based upon these observations, it appears that the extent of codon usage bias in these genes is generally constant. The ENC values were analyzed for possible correlations with the nucleotide compositional parameters, particularly GC_3_ content which has been shown previously to correlate with the former [12]. The results of our analyses are in accordance with the significant positive correlations between ENC and GC_3_ (*r* = 0.431, *P* = 0.014) as well as ENC and overall GC content (*r* = 0.724, *P* = 0.0001).

### 3.2. Characteristics of Synonymous Codon Usage

In an attempt to find out the nature of codon usage bias in the genes under study, the RSCU values of the 59 codons were analyzed (Table 3). Interestingly, most of the preferred codons ended with nucleotide* A*. Among the preferred codons, dinucleotide CpG is markedly suppressed while dinucleotides TpA and CpA were found to be abundant in most of them.

**Figure 2:**
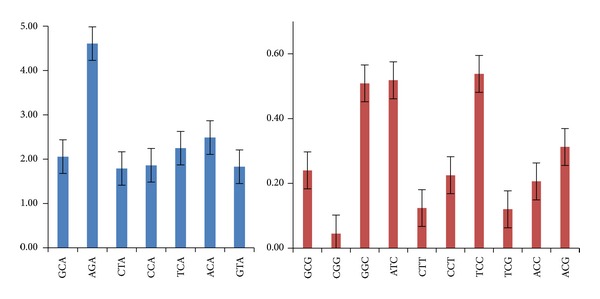
Over- and underrepresented codons in the genes used in the study. The overrepresented codons (RSCU > 1.6) are shown in blue, while the underrepresented (RSCU < 0.6) ones are shown in red.

In quest for possible under- and over-representation of codons, RSCU values were sorted from lower to higher values. We observed that majority of the codons, both preferred as well as non-preferred, fall under unbiased or randomly used category (0.6 < RSCU < 1.6). Seven codons (GCA, AGA, CTA, TCA, ACA and GTA) showed very high RSCU values (RSCU > 1.6) and hence, were considered to be “over-represented”. Similarly there were ten under-represented codons (RSCU < 0.6) (Figure 2).

**Figure 3:**
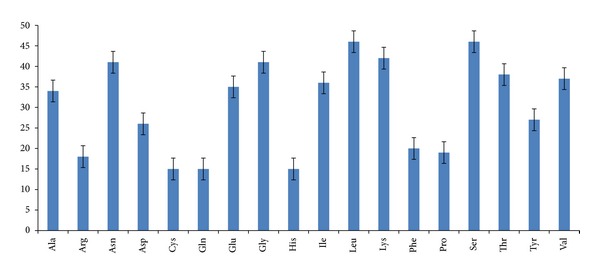
Frequency of the amino acid usage in the genes under study. Leucine and serine are clearly the most frequent amino acids.

**Figure 4:**
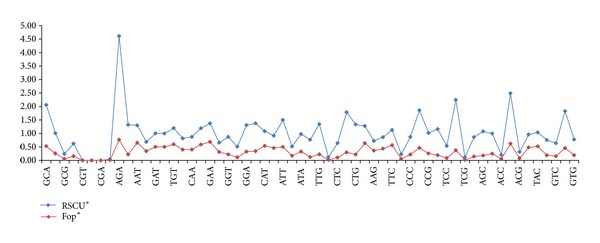
Trend of RSCU and Fop values in the coding sequences of the genes.

All the amino acids showed preference over a particular codon except* Asp* where both the codons were used equally (Figure 4). Surprisingly, in all the accessions, out of six possible codons for* Arg,* only two codons,* namely,* AGA and AGG, were used omitting the rest four. Among these two codons, there was a high bias towards AGA with RSCU values 4.61 as compared to that of 1.32 for AGG.* Ser* and* Leu* were the most frequently used amino acids, while* Cys*,* Gln* and* His* were used least frequently. Frequency of the amino acids* Lys*,* Gly*,* Asn*,* Thr*,* Val* etc. were also towards higher side (Figure 3).

Highly expressed genes show a tendency of high biasness towards some codons and tend to use those codons frequently. To find out such biasness and predict the expression of the genes, CAI values were estimated, values of which range from 0 to 1. The CAI values for the hemagglutinin genes were found in the range of 0.3143–0.3447 with an average of 0.3829 and standard deviation of 0.0391, indicating that the codons are not translationally optimized for expression of these genes.

The frequency of optimal codons (Fop) in a gene can be used as an indicative measure to check if the codons are optimized for efficient translation [23]. The optimized codons refer to the codons with highest transfer RNA (tRNA) copy number. The results showed a similar trend of Fop to that of RSCU values; the codons with higher RSCU values also tend to have higher Fop values (Figure 4). These two parameters showed a significant positive correlation with correlation coefficient of *r* = 0.710 (*P* = 0.0001).

## 4. Conclusion

Amidst much debate, mutational pressure and natural selection have been cited as the major stimulants in framing the codon usage profiles of different viruses [5, 20, 24]. As in most of the RNA viruses, mutation rate of IAV is very high and the effects of codon usage bias are too small for natural selection to act effectively [25]. One possible explanation for lower codon preferences might be due to the fact that it helps the virus to replicate readily in alternate hosts with different codon choices [5].

Hemagglutinin constitutes one of the most important sites for human immune system to act on, thus, making it a potential drug target against this virus. Untangling the underlying mechanisms operating behind the synonymous codon usage profile of the virus will possibly bring up new avenues in the research involving development of antiviral drugs against this hazardous virus.

## Supplementary Material

Table shows information regarding the coding sequences (CDS) of the H1N1 hemagglutinin (HA) genes used to carry out the present study. A total of 32 CDS were used in the study. The sequences were retrieved from Genebank whose accession numbers and the length of the respective CDS are presented in the table.

## Figures and Tables

**Table 1:** Nucleotide composition of the genes used in the study.

Sl No.	A%	T%	G%	C%	A_3_%	T_3_%	G_3_%	C_3_%	GC%	GC_3_%	AT%	AT_3_%	ENC
1	35.4	24.2	22	18.3	36.4	27	16.6	20	40.3	38.4	59.7	61.6	57
2	35.2	23.9	22.3	18.6	35.5	26	17.5	20.9	40.9	40.2	59.1	59.8	58
3	35.4	24.2	22.2	18.2	36.6	27	16.4	20	40.4	38.3	59.6	61.7	57
4	35.2	23.9	22.3	18.6	35.3	26	17.7	20.9	40.9	40.4	59.1	59.6	58
5	35	24	22.4	18.6	35.3	26	17.7	20.9	41	40.4	59	59.6	58
6	35.3	24	22.2	18.4	36.5	26.6	16.4	20.4	40.6	38.8	59.4	61.2	58
7	35.3	23.9	22.3	18.5	35.7	26	17.3	20.9	40.8	40	59.2	60	58
8	35.4	23.9	22.2	18.5	35.5	26.2	17.5	20.8	40.7	40	59.3	60	58
9	35.4	24.2	22	18.3	36.7	27	16.2	20.1	40.4	38.3	59.6	61.7	57
10	35.4	24.2	22.2	18.2	36.7	27	16.2	20.1	40.4	38.3	59.6	61.7	57
11	35.6	24.2	21.9	18.4	37.2	26.6	15.8	20.4	40.3	38.6	59.7	61.9	57
12	35.3	23.9	22	18.8	36.1	25.9	16.6	21.4	40.8	39.9	59.2	60.1	58
13	35.2	23.8	22.2	18.8	35.9	25.5	17.1	21.5	41	40.4	59	59.6	58
14	35.3	24	22.2	18.4	36.5	26.6	16.2	20.6	40.6	39	59.4	61.2	58
15	35.4	24	22.2	18.5	36.4	26.1	16.8	20.7	40.6	38.8	59.4	61	58
16	34.9	24.1	22.4	18.6	36	26.1	17.1	20.8	41	39.2	59	60	58
17	35.3	24	22.1	18.5	36.7	26.2	16.2	20.9	40.7	38.7	59.3	60.9	58
18	35.3	24.2	22.1	18.4	36.4	26.4	16.6	20.7	40.6	38.7	59.4	60.8	58
19	35.1	24.1	22.4	18.4	36.4	26.5	16.5	20.5	40.8	38.8	59.2	61	58
20	35	24.2	22.2	18.6	36.4	25.9	16.7	21	40.8	38.6	59.2	60.2	58
21	35.3	24.1	22.2	18.5	36.4	26.5	16.4	20.7	40.6	38.7	59.4	61	58
22	35.1	24.1	22.4	18.3	36.2	26.7	16.7	20.4	40.7	38.8	59.3	61	58
23	35.1	24.2	22	18.6	36.3	26.3	16.3	21.3	40.7	38.7	59.3	60.4	58
24	35.3	24.1	22.2	18.5	36.4	26.4	16.5	20.7	40.6	38.5	59.4	60.8	58
25	35.2	24	22.2	18.5	36.3	26.3	16.3	21.1	40.8	38.6	59.2	60.7	58
26	35.2	24	22.3	18.5	36	26.4	16.7	20.9	40.8	38.7	59.2	60.5	58
27	34.9	24	22.3	18.7	36.3	25.9	16.8	20.9	41	39	59	60.2	58
28	35.3	24	22.3	18.5	36.4	26.4	16.5	20.7	40.7	38.6	59.3	60.8	58
29	35	24.2	22.2	18.6	36.6	26.1	16.4	20.8	40.8	39	59.2	60.4	58
30	35.5	24	22.2	18.3	37	26.3	16.3	20.3	40.4	38.6	59.6	61.4	58
31	35.3	24	22.2	18.5	36.6	26.2	16.6	20.6	40.7	39.2	59.3	61.8	58
32	35.4	23.8	22.1	18.7	36.4	26	16.9	20.7	40.8	38.9	59.2	60.7	58

**Table 2:** Correlation between different nucleotide compositional parameters.

	A_3_%	T_3_%	G_3_%	C_3_%	GC_3_%	AT_3_%
A%	*r* = 0.425∗	*r* = 0.444∗	*r* = −0.366∗	*r* = −0.471∗∗	*r* = −0.264^∧^	*r* = 0.613∗∗
T%	*r* = 0.539∗∗	*r* = 0.653∗∗	*r* = −0.577∗∗	*r* = 0.512∗∗	*r* = −0.695∗∗	*r* = 0.537∗∗
G%	*r* = −0.515∗∗	*r* = −0.241^∧^	*r* = 0.542∗∗	*r* = 0.089^∧^	*r* = 0.329^∧^	*r* = −0.426∗∗
C%	*r* = −0.478∗∗	*r* = −0.899∗∗	*r* = 0.468∗∗	*r* = 0.883∗∗	*r* = 0.618∗∗	*r* = −0.776∗∗
GC%	*r* = −0.693∗∗	*r* = −0.810∗∗	*r* = 0.664∗∗	*r* = 0.767∗∗	*r* = 0.669∗∗	*r* = −0.886∗∗
AT%	*r* = 0.693∗∗	*r* = 0.810∗∗	*r* = −0.664∗∗	*r* = −0.767∗∗	*r* = −0.669∗∗	*r* = 0.886∗∗

*Means correlation is significant at the level of 0.05.

∗∗Means correlation is significant at the level of 0.001.

^∧^Means no correlation.

**Table 3:** Synonymous codon usage pattern in 32 coding sequences.

AA	Codon	RSCU∗	Fop∗	N*
Ala	**GCA**	**2.06**	**0.53**	**18**
	GCC	1.01	0.26	9
	GCG	0.24	0.06	2
	GCT	0.62	0.16	5

Arg	CGT	0.00	0.00	0
	CGC	0.00	0.00	0
	CGA	0.00	0.00	0
	CGG	0.05	0.01	0
	**AGA**	**4.61**	**0.77**	**14**
	AGG	1.32	0.22	4

Asn	**AAT**	**1.30**	**0.66**	**27**
	AAC	0.69	0.34	14

Asp	GAT	1.00	0.50	13
	GAC	1.00	0.50	13

Cys	**TGT**	**1.20**	**0.60**	**9**
	TGC	0.81	0.40	6

Gln	CAA	0.87	0.40	6
	**CAG**	**1.19**	**0.59**	**9**

Glu	**GAA**	**1.38**	**0.69**	**24**
	GAG	0.66	0.31	11

Gly	GGT	0.87	0.22	9
	GGC	0.51	0.12	5
	GGA	1.31	0.33	13
	**GGG**	**1.37**	**0.34**	**14**

His	**CAT**	**1.08**	**0.54**	**8**
	CAC	0.92	0.46	7

Ile	**ATT**	**1.50**	**0.50**	**18**
	ATC	0.52	0.17	6
	ATA	0.98	0.33	12

Leu	TTA	0.77	0.12	6
	TTG	1.35	0.22	10
	CTT	0.12	0.02	1
	CTC	0.64	0.11	5
	**CTA**	**1.79**	**0.30**	**14**
	CTG	1.33	0.22	10

Lys	**AAA**	**1.28**	**0.64**	**27**
	AAG	0.72	0.36	15

Phe	TTT	0.86	0.44	9
	**TTC**	**1.13**	**0.56**	**11**

Pro	CCT	0.23	0.06	1
	CCC	0.87	0.22	4
	**CCA**	**1.86**	**0.47**	**9**
	CCG	1.02	0.26	5

Ser	TCT	1.16	0.19	9
	TCC	0.54	0.09	4
	**TCA**	**2.25**	**0.37**	**17**
	TCG	0.12	0.02	1
	AGT	0.87	0.14	7
	AGC	1.08	0.18	8

Thr	ACT	1.00	0.25	9
	ACC	0.21	0.05	2
	**ACA**	**2.49**	**0.62**	**24**
	ACG	0.31	0.08	3

Tyr	TAT	0.96	0.48	13
	**TAC**	**1.04**	**0.52**	**14**

Val	GTT	0.76	0.19	7
	GTC	0.64	0.16	6
	**GTA**	**1.83**	**0.46**	**17**
	GTG	0.78	0.19	7

Note: ∗All values are mean values; *N* represents the number of codons; the preferentially used codons for each amino acid are described in bold.
