# Supplementary material for: Compositional Constraint Is the Key Force in Shaping Codon Usage Bias in Hemagglutinin Gene in H1N1 Subtype of Influenza A Virus
Source: Int J Genomics. 2014 Jul 17;2014:349139. doi: 10.1155/2014/349139 (PMC4124814; doi:10.1155/2014/349139)
Supplement: Supplementary file 1 — Table shows information regarding the coding sequences (CDS) of the H1N1 hemagglutinin (HA) genes used to carry out the present study. A total of 32 CDS were used in the study. The sequences were retrieved from Genebank whose accession numbers and the length of the respective CDS are presented in the table. [file 349139.f1.pdf]

Supplementary table 1.

Information of the coding sequences of hemagglutinin (HA) genes used in the study

Sl No	Accession No	Length
**1**	KF280655	1701
**2**	KF280663	1701
**3**	KF280671	1701
**4**	KF280679	1701
**5**	KF280687	1701
**6**	KF280695	1701
**7**	KF280703	1701
**8**	KF280711	1701
**9**	KF280719	1701
**10**	KF280727	1701
**11**	KF280735	1701
**12**	KF280743	1701
**13**	KF280751	1701
**14**	JQ319658	1701
**15**	JQ319657	1701
**16**	HM204579	1770
**17**	HM204578	1743
**18**	HM204577	1743
**19**	HM204576	1707
**20**	HM204575	1770
**21**	HM204574	1707
**22**	HM204573	1707
**23**	HM204572	1743
**24**	HM204571	1707
**25**	HM204570	1710
**26**	HM204569	1707
**27**	HM204568	1755
**28**	HM204567	1707
**29**	HM204566	1767
**30**	JN600356	1701
**31**	JF293316	1701
**32**	JF293315	1701
